# Chemical Speciation and Bond Lengths of Organic Solutes by Core-Level Spectroscopy: pH and Solvent Influence on *p*-Aminobenzoic Acid

**DOI:** 10.1002/chem.201405635

**Published:** 2015-03-18

**Authors:** Joanna S Stevens, Adrian Gainar, Edlira Suljoti, Jie Xiao, Ronny Golnak, Emad F Aziz, Sven L M Schroeder

**Affiliations:** [a] School of Chemical Engineering and Analytical Science, School of Chemistry, The University of Manchester Oxford Road, Manchester M13 9PL (UK) E-mail: slm.schroeder@outlook.com; [b] Joint Ultrafast Dynamics Lab in Solutions and at Interfaces (JULiq), Institute of Methods for Material Development, Helmholtz-Zentrum Berlin für Materialien und Energie Albert Einstein Strasse 15, 12489 Berlin (Germany); [c] Fachbereich Physik, Freie Universität Berlin Arnimallee 14, 14195 Berlin (Germany); [d] School of Chemical and Process Engineering, University of Leeds Leeds LS2 9JT (UK)

**Keywords:** ionization potentials, liquids, X-ray scattering, speciation, X-ray absorption spectroscopy

## Abstract

Through X-ray absorption and emission spectroscopies, the chemical, electronic and structural properties of organic species in solution can be observed. Near-edge X-ray absorption fine structure (NEXAFS) and resonant inelastic X-ray scattering (RIXS) measurements at the nitrogen K-edge of *para*-aminobenzoic acid reveal both pH- and solvent-dependent variations in the ionisation potential (IP), 1s→π* resonances and HOMO–LUMO gap. These changes unequivocally identify the chemical species (neutral, cationic or anionic) present in solution. It is shown how this incisive chemical state sensitivity is further enhanced by the possibility of quantitative bond length determination, based on the analysis of chemical shifts in IPs and σ* shape resonances in the NEXAFS spectra. This provides experimental access to detecting even minor variations in the molecular structure of solutes in solution, thereby providing an avenue to examining computational predictions of solute properties and solute–solvent interactions.

## Introduction

The ability to observe chemical and structural changes in organic molecules in solution is of central importance for progress in chemical and life sciences. For example, the activity of biological systems is strongly influenced by pH variations causing changes in chemical speciation,[1] whereas the structural outcomes of self-assembly and crystallisation processes define physical and chemical properties of products.[2, 3] In particular, details of the structural evolution of nucleating solutions are of contemporary interest[3, 4] because of their far-reaching impact on both structural and morphological outcomes, the tailoring of product properties and the design of materials.

A crucial prerequisite for further elucidation of molecular structure in such processes is having the experimental capability to incisively study conformational structure, bonding and electronic properties of organic molecular solutes in situ.[3, 5] Core-level spectroscopies are emerging as suitable probes for this task, as they are highly sensitive to local electronic structure and bonding. Even X-ray photoelectron spectroscopy (XPS),[6] one of the most facile core-level spectroscopies in the laboratory, permits the characterisation of protonation and hydrogen bonding in both solid-state and solution acid–base systems through determination of chemical shifts in core-level binding energies.[7, 8]

Near-edge X-ray absorption fine structure (NEXAFS), also known as X-ray absorption near-edge structure (XANES), is chemically and structurally much more incisive,[9, 10] as it involves the excitation of core electrons to unoccupied valence orbitals that are readily interpreted by use of molecular orbital (MO) calculations. In addition, σ* shape resonances in the NEXAFS are very sensitive to bond length variations, thus providing incisive molecular structure parameters, even for non-crystalline systems.[11] Resonant inelastic X-ray scattering (RIXS) probes occupied valence orbitals when the correlation of the valence electrons is weak, by monitoring the transitions of electrons from occupied valence orbitals into core-hole states.[12] The combination of NEXAFS and RIXS thus provides a comprehensive picture of the local electronic structure and is ideally placed for investigations of local environment and bonding of molecular solutes. Recent development of the liquid microjet technique[13, 14] allows windowless in situ probing of solute species in solution and, importantly for organics, alleviates potential problems caused by radiation damage. In addition, the ultra-fast nature of the electronic transitions (≈10^−15^ s)[9] means that every detected event represents a snapshot of a molecule rather than an average over vibrational or conformational changes, thereby permitting population analysis when transient species coexist.[8, 15]

**Figure 1:**
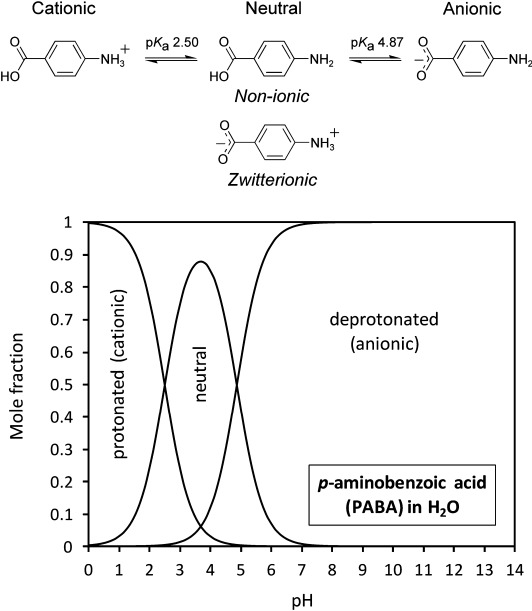
Equilibria between the cationic, neutral and anionic PABA species in water (top) and speciation diagram as a function of pH (bottom).

Herein, we demonstrate how NEXAFS and RIXS microjet studies can be combined with density functional theory (DFT) calculations to elucidate the pH- and solvent-dependent changes in the local chemical and electronic environment of *para*-aminobenzoic acid (PABA, Figure 1). PABA is an intermediate for folic acid synthesis and has pharmaceutical applications.[16] Depending on pH and solvent it forms neutral, zwitterionic, cationic and anionic species in solution.[17, 18] We will show how direct observation of these chemically and electronically distinct species is possible by monitoring the nitrogen 1s core-level excitation (nitrogen K-edge), thereby providing electronic structure signatures for aqueous solutions with varying pH as well as for alcoholic solutions. We will correlate electronic structure and bond length changes with the effects of ionisation at Brønsted donor and acceptor moieties, and compare the results with data for non-ionic species in the solid state and DFT molecular orbitals (MOs).

## Results and Discussion

### Expected speciation in solution and in the solid state

The solubility of PABA in aqueous solution is very low (0.03 m) and dissolution leads to a complex mixture at the isoelectric point (pH 3.69), which comprises non-ionic and zwitterionic neutral species[17] alongside small percentages of cationic and anionic species (Figure 1).[19] Based on the p*K*_a_ values,[19] upon increasing the pH above 4.87 the carboxylate anionic form dominates, whereas decreasing the pH below 2.50 results in solutions containing almost entirely the cationic form (Figure 1). These ionic forms have greatly increased solubility (>0.5 m) and allow microjet NEXAFS and RIXS measurements to be recorded with good signal-to-noise ratio. In the solid state, PABA exists as neutral, non-zwitterionic molecules bonded through a series of intermolecular hydrogen bonds and π–π interactions,[20–22] thus providing a spectrum of the non-ionic form for comparison with the ionic species expected at high and low pH values. For comparison we have also examined alcoholic solutions, in which PABA is expected to exist primarily in the non-ionic neutral form (i.e., non-zwitterionic).[17]

### NEXAFS

**Figure 2:**
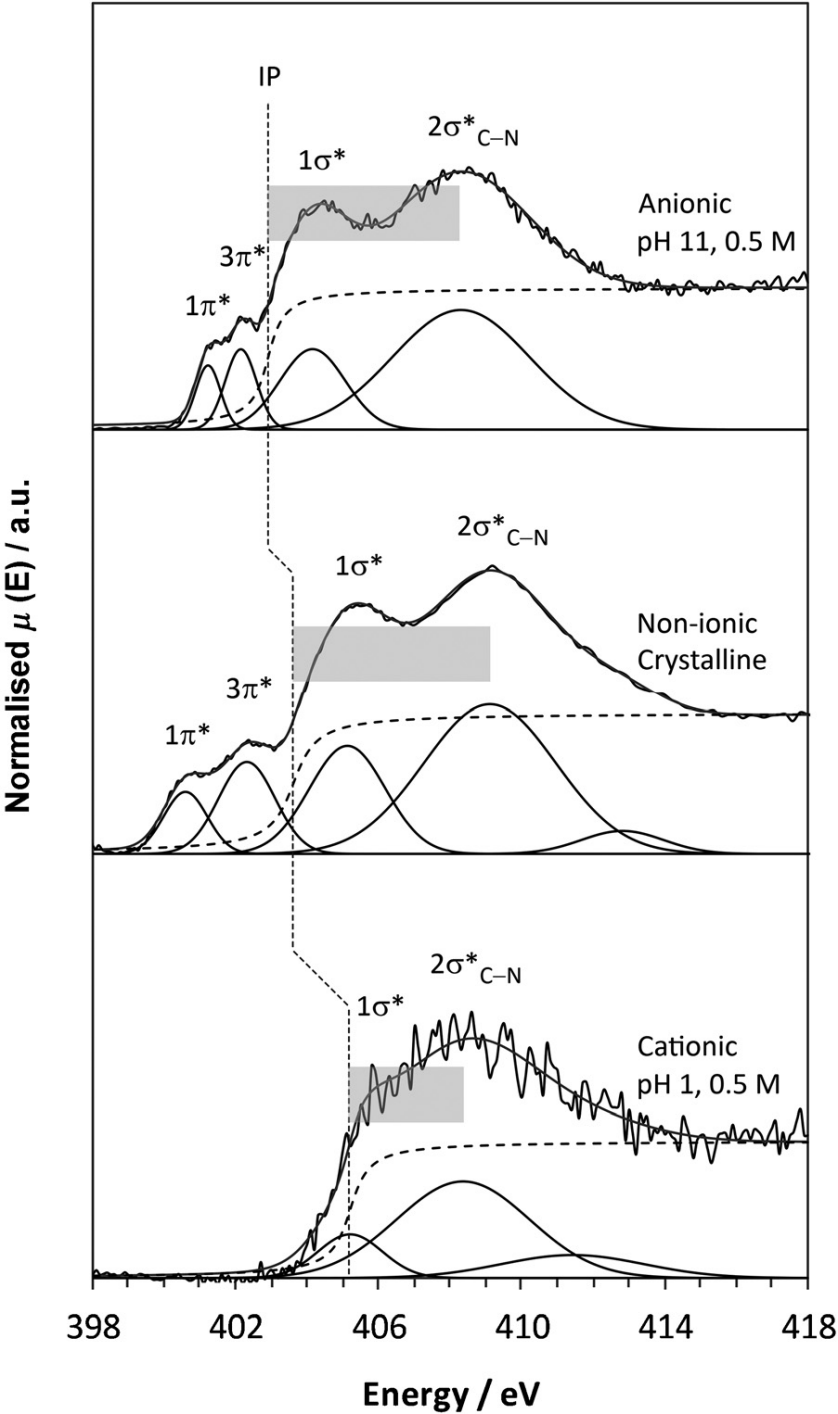
Nitrogen K-edge NEXAFS spectra for anionic (top), non-ionic solid-state β- (middle) and cationic PABA (bottom), together with the fits to the data; IPs are marked by vertical dashed lines, and the magnitude of the term value *δ*_C–N_ is indicated by shaded boxes.

Comparison of the nitrogen K-edge NEXAFS spectra for non-ionic, solid-state PABA and in situ measurements at high/low pH in aqueous solution (Figure 2) reveals marked differences between the electronic structure of the species at pH 11 and pH 1, as well as variations between the non-ionic and pH 11 forms. Two pre-edge resonances (energetically below the ionisation potential, IP) are evident for the non-ionic form and at pH 11, which arise from 1s→π* transitions due to conjugation of the nitrogen lone pair with the aromatic ring, and these are followed by the broader 1s→σ* resonances that are more susceptible to variations in geometric structure.[9]

The IP for the species present at pH 1 is very different from that observed at pH 11, with a positive energy shift of 2.3 eV (Figure 2, Table 1). This increased IP for the pH 1 solution species reflects acquisition of a positive charge on nitrogen for the cationic form (decreased electron density, orbital contraction). A slight decrease in IP occurs at pH 11 compared to the non-ionic form (Table 1), with conjugation of the carboxyl and amine groups through the aromatic ring leading to some orbital screening and redistribution of electron density occurring with change to the anionic form (Figure 1). Due to the absence of the lone electron pair on the nitrogen atom with NH_3_^+^, the cationic nitrogen will not be able to participate in the π MOs with the aromatic ring; therefore, no pre-edge π* peaks are visible in the pH 1 nitrogen K-edge (Figure 2), further signifying transformation to the cationic form. Interestingly, the π* resonances become closer to one another in the anionic form at pH 11 compared to the non-ionic form, which leads to a smaller Δπ* value for the anionic species (Figure 2, Table 1).

**Table 1:** Experimental nitrogen K-edge NEXAFS resonance and IP energies from Figure 2 along with the term value *δ*_C–N_

Nitrogen [eV]	Non-ionic β-PABA	Anionic pH 11	Cationic pH 1	Non-ionic methanol
IP	403.60	402.90	405.20	403.47
1π^*^	400.58	401.22	–	400.68
3π^*^	402.30	402.14	–	401.98
1σ^*^_N–H_	405.11	404.15	405.20	403.66
2σ^*^_C–N_	409.09	408.30	408.36	408.66
Δπ^*^	1.72	0.92	–	1.30
*δ*_C–N_ (σ^*^_C–N_−IP)	5.49	5.40	3.16	5.19

**Figure 3:**
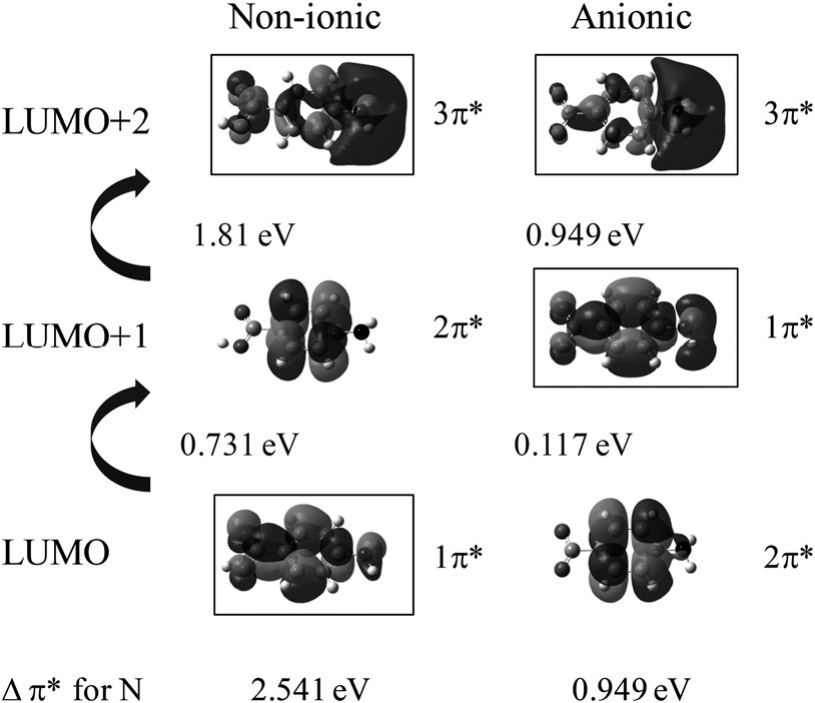
Lowest unoccupied molecular orbitals (LUMOs) for the non-ionic and anionic monomers of PABA. The π* orbitals are labelled 1π*, 2π* and 3π* from the lowest energy relative to non-ionic PABA and those with weight on the nitrogen absorber are marked with boxes; transitions from the N 1s core level to these marked orbitals are visible in the nitrogen K-edge NEXAFS.

DFT calculations of the unoccupied MOs with nitrogen contributions allow us to pinpoint the electronic transitions leading to pre-edge π* nitrogen intensity in the anionic and non-ionic NEXAFS and to interpret the magnitude of the calculated energy shifts. Figure 3 shows the lowest unoccupied molecular orbitals (LUMOs) for non-ionic and anionic PABA, which reveals that the calculated gap between the two lowest-energy nitrogen π* orbitals (Δπ*) is over twice as large for non-ionic PABA as for the anionic form. In addition, the π* energies for anionic PABA are raised above that for PABA, with the nitrogen 1π* becoming the LUMO+1 rather than the LUMO (Figure 3).

**Figure 4:**
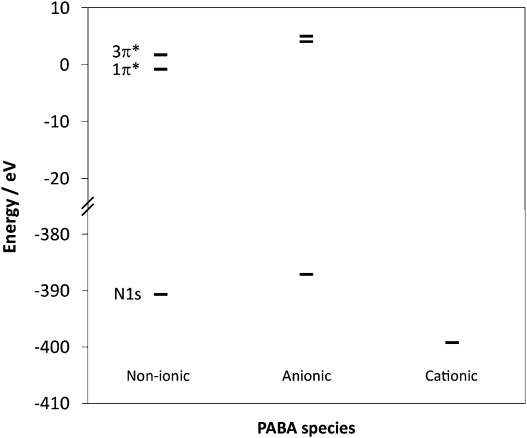
Calculated orbital energies for PABA showing the trends in nitrogen π* (top) and core 1s (bottom) orbital energies for non-ionic, anionic and cationic forms.

The experimental trend in the IP (Figure 2) for the different PABA species is also reproduced by the calculations (Figure 4), with a decrease in core-level N 1s energy for cationic species (thus increased IP) and a slight increase for anionic relative to non-ionic PABA (decreased IP). The nitrogen π* LUMOs for the anionic form mirror the change in the core-level energy, with an increase in energy relative to non-ionic PABA (Figure 4). The energy gap between the 1π* and 3π* MOs also narrows for the anionic form (Figures 3 and 4); this decrease in the gap between the nitrogen π* resonances (Δπ*) for the anionic form parallels that observed in the experimental NEXAFS (Figure 2), explaining it by a significant increase in the 1s→1π* energy and small decrease in the 1s→3π* energy relative to non-ionic PABA occurring with formation of the carboxylate form (Table 2). Comparison of calculations for the zwitterionic form (Table 2) illustrate that its presence would significantly raise the IP, albeit perhaps not quite as much as for the cationic form, alongside the loss of resolved pre-edge π* peaks through the positive charge on the nitrogen. Although the magnitude of the calculated energy shifts may not be accurate due to the absence of core-hole relaxation and screening from intermolecular interactions, the direction and trends observed correlate well with the experimental data, thus indicating that variations in ground-state (initial-state) properties allow the interpretation of electronic structure probed through core-level excitation, which tends to dominate over final-state effects.

**Table 2:** Calculated orbital energies and shifts for different PABA species

For N [eV]	Non-ionic	Anionic	Cationic	Zwitterionic
N 1s	−390.66	−387.10	−399.24	−396.02
N 1s→1π^*^	389.86	391.16	–	–
N 1s→3π^*^	392.40	392.11	–	–
Δπ^*^ (1π^*^→3π^*^)	2.54	0.95	–	–

### C–N bond lengths

**Figure 5:**
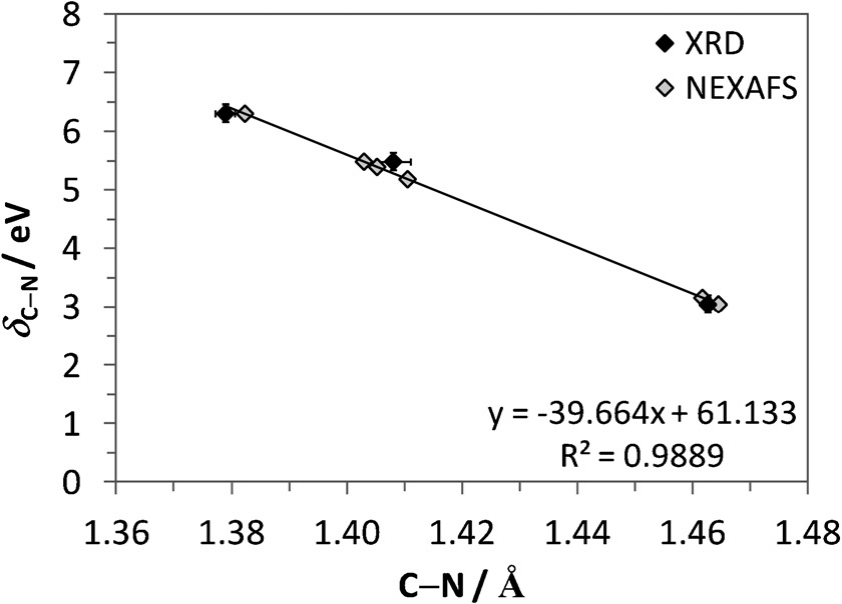
Correlation between the NEXAFS term value (*δ*_C–N_) and C–N bond lengths from XRD (black),[20, 22, 24] and comparison with C–N bond lengths obtained from NEXAFS (grey) for the non-ionic and ionic PABA species (Table 3).

The experimental nitrogen K-edge NEXAFS spectra (Figure 2) also show variation in the position of the broader, post-edge σ* shape resonances relative to the IP for the non-ionic, anionic and cationic species. For a bond between two atoms X and Y, the term value *δ*_X–Y_, the difference between the energy of its σ*_X–Y_ orbital and the IP of either X or Y, provides a highly sensitive measure of the bond length.[9, 23] As can be seen in Figure 2, and from the term values (*δ*_C–N_) in Table 1, the separation between the energy of the σ*_C–N_ resonance and the IP is of a similar magnitude for anionic and non-ionic PABA. In contrast, the energy of σ*_C–N_ and the IP are much closer for the cationic species, which reflects an increase in IP and decrease in the energy of the σ*_C–N_ resonance following protonation of the amino group, and results in a substantially smaller *δ*_C–N_ value (Figure 2). As the C–N bond lengths are known from X-ray diffraction (XRD) crystal structures for the solid state (β non-ionic form[22] and cationic HCl salt,[24] as well as the additional non-ionic α form[20] previously investigated,[25] Table 3), these can be used to obtain a calibration plot for bond length determination in the solutions. Plotting the term value *δ*_C–N_ against the C–N bond lengths in the corresponding crystal structures indicates a linear dependence (Figure 5, black data points). The correlation between XRD-derived bond lengths and NEXAFS-derived term values is very high (Figure 5), which suggests sensitivity to bond length variations of less than 0.005 Å (Table 3). The term values for the solution species are similar to those found in their corresponding solid-state structures, thus suggesting that determination of the term values *δ*_C–N_ measures their bond length reliably (Table 3, Figure 5 grey data points) in both the solid state and in solution. We expect some variation in bond lengths as a result of the differences in local environment, for example, due to interaction with solvent molecules. The observed impact on the C–N bond length is on the order of less than tenths of an Ångstrom, but appears to be within the detection limit. Certainly the expected trend, overall longest C–N bonds for protonated nitrogen in the cationic species and shorter bonds for the neutral as well as the anionic species, is evident for both the solid and the liquid phase. This indicates strong potential for a generalisation of the σ* shape resonance analysis to other organic molecular solutes, and even “measuring” bond lengths in solution.

**Table 3:** Experimental C–N bond lengths from XRD and those derived from NEXAFS through *δ*_C–N_

Nitrogen	*δ*_C–N_ [eV]	XRD C–N [Å]	NEXAFS C–N [Å]
non-ionic α-PABA	6.31^[a]^	1.379(2)^[b]^	1.3822
non-ionic β-PABA	5.49	1.408(3)^[c]^	1.4029
anionic pH 11	5.40	[1.410(2)^[d]^]	1.4052
non-ionic methanol	5.19	–	1.4105
cationic pH 1	3.16	–	1.4617
cationic HCl salt	3.05	1.4626(5)^[e]^	1.4644

[a] Ref. [25]. [b] Ref. [20]. [c] Ref. [22]. [d] C–N bond length from the hydrated Na salt crystal structure[26] shown for comparison. [e] Ref. [24].

### Methanol species

**Figure 6:**
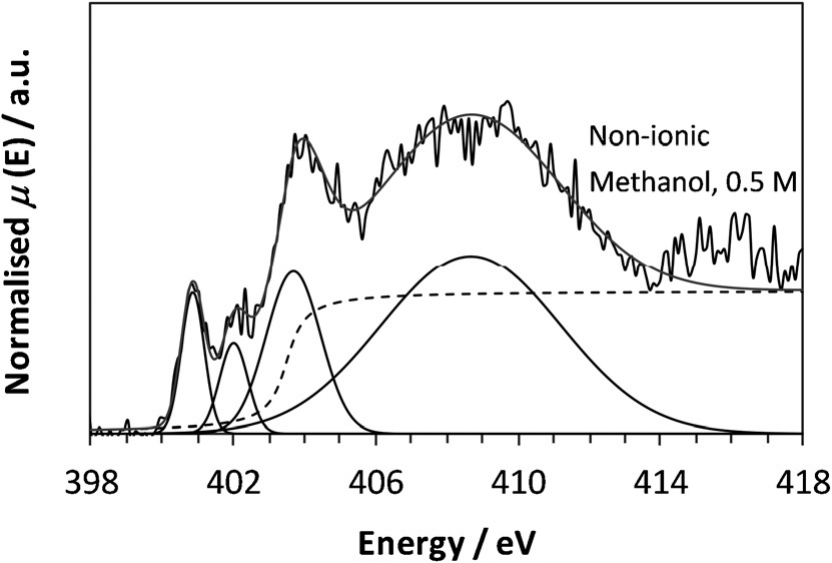
Nitrogen K-edge NEXAFS of PABA in methanol (0.5 m).

Comparison with the nitrogen K-edge NEXAFS for PABA obtained in methanol (Figure 6) reveals a shape in agreement with that of non-protonated nitrogen species (Figure 2). The IP and 1π* resonance (with their previously demonstrated sensitivity to the chemical species present) are consistent with those observed for the non-ionic form rather than anionic species (Table 1). If the molecules in the 0.5 m methanol solution were primarily zwitterionic (Figure 1), a spectrum resembling that of the cationic form (Figure 2) would be expected, with no pre-edge π* resonances and an increased IP resulting from the positive charge on nitrogen. That this is not observed provides direct evidence that (at least the majority of) PABA molecules in methanol exist in the neutral, uncharged (non-ionic) form. The smaller *δ*_C–N_ for the methanol species relative to the solid state (Table 1) suggests that the C–N bond is slightly longer in the methanol solution, and applying the correlation above gives a predicted C–N bond length of 1.4105 Å (Table 3).

### RIXS

**Figure 7:**
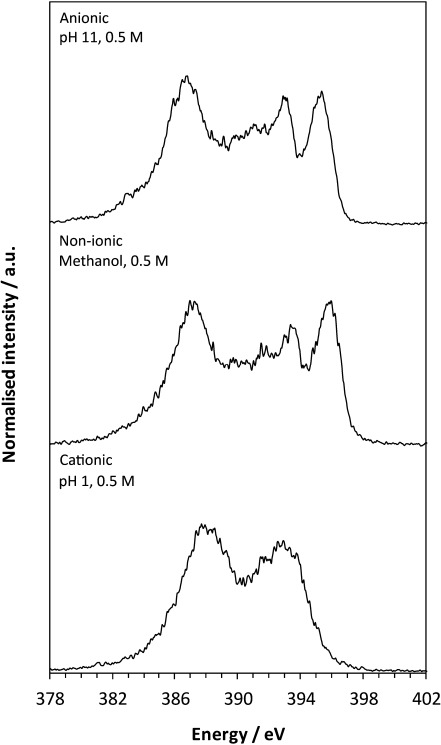
Nitrogen RIXS for anionic, non-ionic and cationic species in solution.

While NEXAFS probes electron transitions from the core level to unoccupied orbitals, RIXS involves transitions from occupied valence orbitals to the core hole after excitation.[9] For nitrogen, this follows the 2p→1s transitions. The nitrogen RIXS spectra for the non-ionic and anionic species (methanol and pH 11 solutions, respectively) initially look similar, whereas that for pH 1 is clearly distinguished (Figure 7). Following protonation of nitrogen for the cationic species at low pH, the highest-energy peaks arising from the occupied 2p π valence orbitals→1s are absent due to the use of the nitrogen lone pair in forming an additional N–H bond and not part of the π MOs.

**Figure 8:**
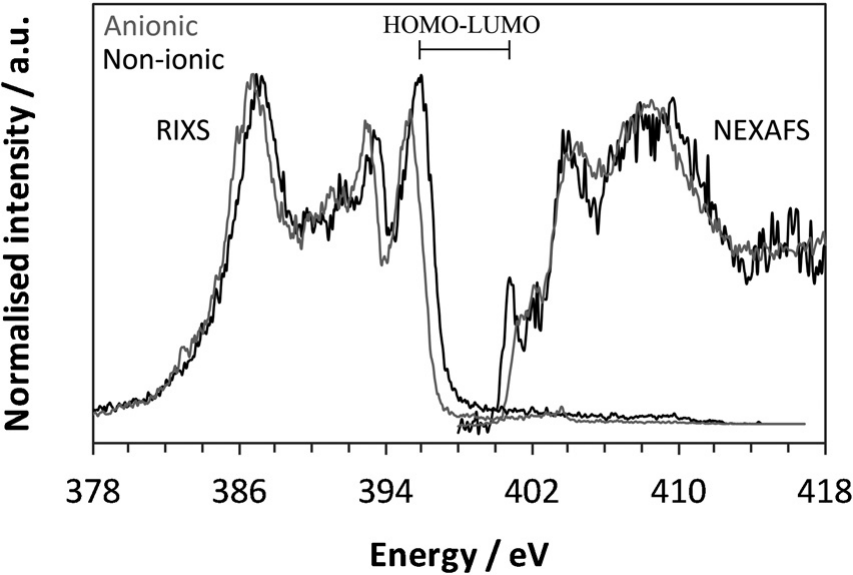
Nitrogen RIXS and NEXAFS showing the shift in energy between anionic and non-ionic solution species and the HOMO–LUMO gap.

**Figure 9:**
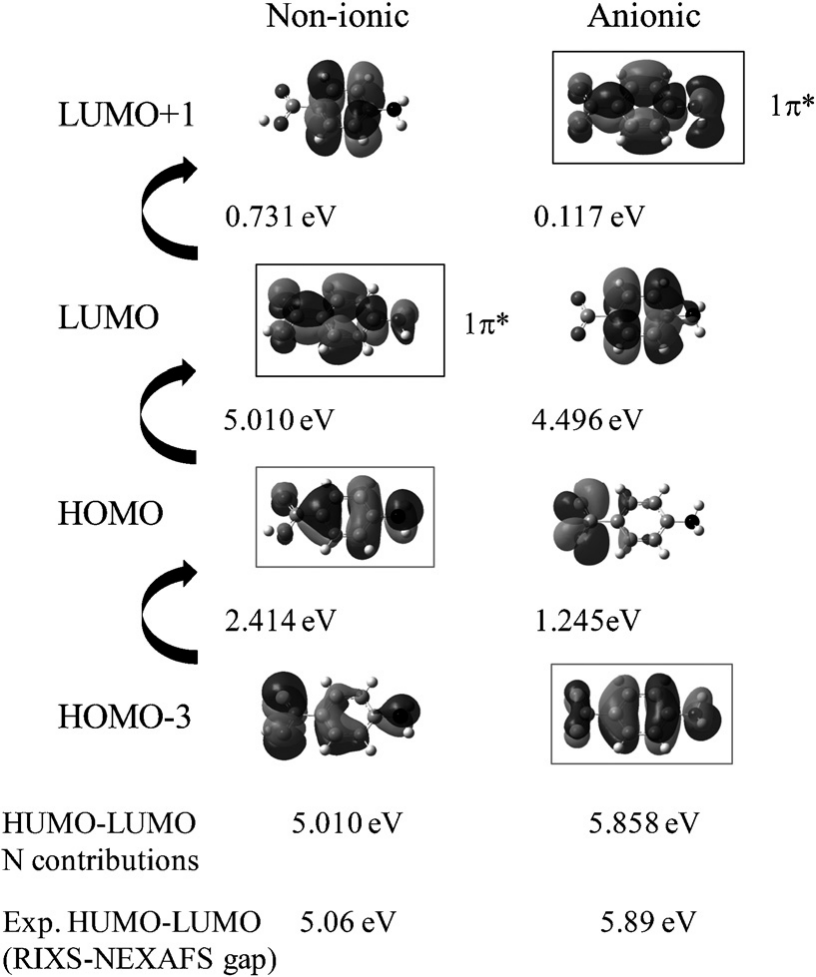
Highest occupied and lowest unoccupied molecular orbitals involved in the first RIXS (grey outline) and NEXAFS (black outline) resonances, respectively, with the experimental and calculated nitrogen HOMO–LUMO gap for non-ionic and anionic PABA.

Closer inspection of the nitrogen RIXS for anionic and non-ionic species reveals a significant shift to lower energy for the anionic form (Figure 8, left). This is particularly noticeable towards the higher-energy region of the RIXS data. As the highest energy (inelastic) peak in the RIXS arises from the decay of electrons from the nitrogen HOMO to the core N 1s level, this indicates a lower energy for this MO for the anionic species. Comparison of the relative energies for the HOMOs with nitrogen contributions for non-ionic and anionic species reveals that the HOMO→1s leads to the first RIXS peak for the non-ionic form (Figure 9). In contrast, this MO is lowered in energy for the anionic form, becoming the HOMO−3 (Figure 9), thereby elucidating the shift to lower energy seen experimentally (Figure 8).

Further insight can be obtained through combination of the RIXS and NEXAFS data (Figure 8), which provides a model of the local density of valence states. The energy difference between the highest-energy RIXS peak and lowest-energy NEXAFS peak is a probe of the gap between the HOMO and LUMO per atomic chemical state (Figure 8); an alternative method for the bandgap is to take the difference of the intersections between the first peak slopes and the backgrounds (tending to underestimate).[27] For nitrogen in the non-ionic form, this represents the HOMO↔LUMO gap, whereas for the anionic form, this is for HOMO−3↔LUMO+1 (Figure 9). Experimentally, this leads to energy gaps of 5.06 and 5.89 eV for the non-ionic and anionic PABA species, respectively, which compares favourably with the predicted values of 5.01 and 5.86 eV.

## Conclusion

Directly monitoring the core-level transitions of the amino group by nitrogen NEXAFS and RIXS as a function of pH successfully characterises the chemical and electronic state of PABA species in aqueous solution. Formation of the cationic species by protonation of the amino group at low pH leads to a significant shift in IP to higher energy, along with an absence of π* resonances in NEXAFS and of π valence peaks in RIXS. Although the amino group is not protonated in the anionic and non-ionic forms, differences are observed in both the NEXAFS and RIXS. There is a slight shift to low energy for the IP and a widening of the HOMO–LUMO gap for the anionic species, in agreement with predictions of DFT calculations. In methanol, the NEXAFS resembles that of non-ionic PABA, with no indications of the presence of the zwitterionic form. Structural as well as chemical and electronic changes impact the NEXAFS spectra, with variation of the C–N bond length influencing the energy of the σ*_C–N_ resonance relative to the IP, thereby providing access to bond length determination of solutes in solution by comparison with NEXAFS data of PABA species in the solid state.

## Experimental Section

### Solid-state NEXAFS

The solid-state PABA sample (β-PABA[22]) was formed through an aqueous slurry of the commercial form (>99 %, Sigma–Aldrich, UK) at 5 °C. Solid-state NEXAFS measurements were performed at the U7A beamline of the National Synchrotron Light Source (NSLS) at Brookhaven National Laboratory, NY. Partial electron yield (PEY) spectra for the nitrogen K-edge were collected by a channeltron electron multiplier with the sample at the magic angle (54.7°) relative to the incident beam. An entrance grid bias of −150 V was used for PEY collection and a monochromator with a 600 L mm^−1^ grating, which provided energy resolution of about 0.15 eV. After collection, the spectra were normalised by the simultaneously recorded drain current from an in situ gold-coated, 90 % transmission grid (*I*_0_) placed in the incident X-ray beam to eliminate the effect of incident beam intensity fluctuations and beamline optics absorption features, and the monochromator energy was calibrated using the 400.6 eV first π* feature of a titanium nitride grid.

Peak fitting and normalisation was performed using the Athena software,[28] with arctan steps for the IPs (edge steps) and Gaussian functions for the peaks.[9, 25, 29] For investigation of the relationship of C–N bond length with the NEXAFS term value *δ*_C–N_ (σ*_C–N_−IP), the standard deviation for the C–N bond lengths from single-crystal X-ray diffraction (XRD) was ≤0.003 Å[20, 22, 24] and good correspondence was observed for the fitted IP energy shifts with the XPS N 1s core binding energies (≤0.15 eV standard deviation).[25] The correlation between the nitrogen NEXAFS and C–N bond length revealed that a 0.1 eV change in the term value *δ*_C–N_ corresponds to a 0.0025 Å alteration in C–N bond length.

### Solution-state in situ NEXAFS

Solution-state nitrogen K-edge spectra were recorded with the LiXEdrom endstation[14] at the U41-PGM beamline of the BESSY II synchrotron at Helmholtz Zentrum Berlin (HZB), by using the liquid microjet technique. The 0.5 m acidic (HCl) and basic (NaOH) aqueous solutions and 0.5 m methanol solution of PABA were prepared and filtered at ambient pressure to remove impurities or undissolved crystals. Partial fluorescence yield data were recorded in scanning mode by a grating with line density 1200 lines mm^−1^ and radius 7.5 m dispersing the emitted photon energy from the sample,[14] and subsequently a detector consisting of a charge-coupled device, fluorescence screen and microchannel plate stack collecting the amplified signal. The sample, grating and detector were arranged in Rowland circle geometry for accurate focusing. (Although total fluorescence yield could be recorded with a GaAsP photodiode mounted in the vicinity of the liquid jet, there were problems with the signal recording and solute could crystallise on the photodiode, thus interfering with the signal.) Use of the microjet ensured fresh sample was probed by the X-ray beam, vastly minimising the potential for any X-ray-induced damage. An 18 μm diameter glass nozzle with 0.6 mL min^−1^ flow rate was used, and the measurement performed around 2 mm from the nozzle within the laminar part of the jet flow (droplets started to form after 3–5 mm, with the resulting frozen residues collected by a liquid nitrogen trap) with 10^−5^ mbar pressure in the main chamber. Beamline energy calibration was performed with N_2_ gas (total electron yield X-ray absorption spectroscopy 1s→2p π transition) and the resolution was ≤0.1 eV.

### Solution-state in situ RIXS

Microjet nitrogen RIXS spectra were recorded at BESSY II[14, 30] by using the same setup and solutions as for the NEXAFS. Nitrogen RIXS data were recorded at multiple excitation energies corresponding to NEXAFS resonances. As the local valence region available for transitions by excited core electrons has predominantly the N 2p character, little valence electron excitation was expected due to the general weak correlation for the p orbital. In line with this, similar spectral features at constant emission energy with varying excitation energy were observed, and only changes in relative intensity occurred across the NEXAFS transition thresholds (excitation energies of 404.5, 410 and 408 eV for the anionic, non-ionic and cationic species, respectively, were used for further analysis).

### DFT calculations

Non-ionic, anionic and cationic PABA monomers were optimised with the B3LYP functional and 6-31G* basis set in Gaussian 09[31] to obtain MOs[32] for the ground state. This permitted identification of electronic structure changes originating from chemical (initial-state) variations.[9] Strictly speaking, NEXAFS interpretation should also include the influence of relaxation effects due to the presence of the core hole, which were expected to affect the final state of the observed electronic transitions. We have recently included such effects in CASTEP calculations for crystalline PABA[25] and found that the inclusion of final-state effects did not alter the MO interpretation of the NEXAFS substantially. The reason for this insensitivity lies in a combination of the strong localised character of core-level excitations and the weakness of intermolecular interactions (with hydrogen bonding dominant) of organic molecules relative to internal covalent bonding and protonation effects. The ground-state calculations used for the interpretation of the NEXAFS are therefore expected to somewhat overestimate the absolute energies associated with the π* transitions, but less so the relative energies and sequence of the unoccupied π* states.
